# Advances in the pathogenesis of psoriasis: from keratinocyte perspective

**DOI:** 10.1038/s41419-022-04523-3

**Published:** 2022-01-24

**Authors:** Xue Zhou, Youdong Chen, Lian Cui, Yuling Shi, Chunyuan Guo

**Affiliations:** 1 Department of Dermatology, Shanghai Skin Disease Hospital, Tongji University School of Medicine, 200443 Shanghai, China; 2 Institute of Psoriasis, Tongji University School of Medicine, 200443 Shanghai, China

**Keywords:** Immunological disorders, Mechanisms of disease

## Abstract

Psoriasis is a complex long-lasting inflammatory skin disease with high prevalence and associated comorbidity. It is characterized by epidermal hyperplasia and dermal infiltration of immune cells. Here, we review the role of keratinocytes in the pathogenesis of psoriasis, focusing on factors relevant to genetics, cytokines and receptors, metabolism, cell signaling, transcription factors, non-coding RNAs, antimicrobial peptides, and proteins with other different functions. The critical role of keratinocytes in initiating and maintaining the inflammatory state suggests the great significance of targeting keratinocytes for the treatment of psoriasis.

## Facts

- Psoriasis is characterized by the excessive proliferation and abnormal differentiation of keratinocytes and infiltration of multiple inflammatory cells.
- Keratinocytes are critical in psoriasis pathogenesis and participate in both the initiation and maintenance phases of psoriasis.
- Various factors such as genetics, cytokines and receptors, metabolism, cell signaling, transcription factors, non-coding RNAs, antimicrobial peptides, etc. modulate the functions of keratinocytes and influence psoriasis.
- Targeting those factors provides promising therapeutic strategies for psoriasis.

## Open questions

- What’s the key factor that modulates keratinocytes in psoriasis?
- What’s the role of keratinocytes in the relapse of psoriasis?
- Can we achieve higher anti-psoriasis efficacy based on treatment targeting keratinocytes more selectively and efficiently?

## Introduction

Psoriasis is a chronic, inflammatory autoimmune skin disease affected by genetic and various environmental factors. It has been recognized as a significant public health burden and is estimated to affect ~125 million people globally and ~2–4% of the population in western countries [1, 2]. Although the mortality rate of psoriasis is low, patients with psoriasis experience a significant impairment in life quality and a tremendous psychosocial burden.

Psoriasis is characterized by epidermal hyperplasia and dermal infiltration of immune cells. The pathogenesis of psoriasis is complicated, which involves the interplay between keratinocytes, immune cells, and other skin-resident cells. Over the last 2 decades, psoriasis has been considered as an immune cell-driven disease, and keratinocytes are just executors to perform the function of immune cells during psoriasis [3]. And the IL-23/IL-17 pathogenic axis is the key to drive psoriasis. Activation of plasmacytoid dendritic (pDCs) promotes myeloid dendritic cells (mDCs) maturation and production of TNF-α, IL-12, and IL-23, which leads to the activation of Th (T helper) 1 and Th17 and subsequent secretion of inflammatory cytokines, such as TNF-α, IL-17, IL-21, and IL-22. Keratinocytes are then activated by these cytokines (especially IL-17) and produce antimicrobial peptides, cytokines, and chemokines, contributing to the amplification of inflammation [1, 4]. Multiple biologics targeting TNF-α, IL-23, and IL-17 have shown tremendous success in the treatment of psoriasis. However, the side effects, safety, loss of efficacy and recurrence after discontinuation of these biologics encourage researchers to explore novel therapeutic strategies. Emerging evidence has shown that keratinocytes could act as a trigger in psoriasis, and would be a promising target for psoriasis treatment [3].

In this extensive review, we aim to discuss the recent advances in the pathogenesis of psoriasis from keratinocyte perspective. We will discuss multiple factors modulating keratinocytes and how keratinocytes are affected and linked to the pathogenesis of psoriasis.

## The role of keratinocytes in psoriasis pathogenesis

**Fig. 1:**
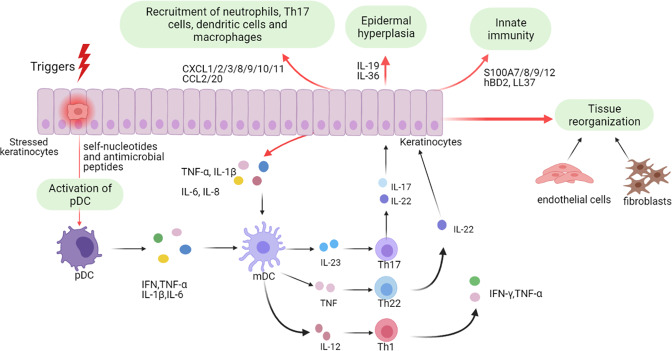
The role of keratinocytes in psoriasis pathogenesis. This figure depicts the pathological process of psoriasis mainly from the keratinocyte perspective. Keratinocytes can be stimulated by initial triggers, and stressed keratinocytes release self-nucleotides and antimicrobial peptide, activate pDCs and subsequent mDCs, involving in the initiation phase of psoriasis. After cytokines stimulation, activated keratinocytes influence psoriasis pathology from aspects of inflammatory infiltration, epidermal hyperplasia, innate immunity, tissue reorganization, etc. pDCs plasmacytoid dendritic cells, mDCs myeloid dendritic cells, IFN interferon, TNF-α tumor necrosis factor-α, IL-1β interleukin-1β, Th1 T helper 1.

Keratinocytes play essential roles in both the initiation and maintenance phases of psoriasis (Fig. 1). As part of the innate immune system, keratinocytes can respond to multiple triggers. Stressed keratinocytes release self-nucleotides and antimicrobial peptides, thus promoting the activation of pDCs. Then mDCs is activated and matured by producing IFN-α, IFN-γ, TNF-α, and IL-1β [5, 6].

Besides participating in the initiation phase, keratinocytes also work as amplifiers of psoriatic inflammation during maintenance phase [7]. Once activated by proinflammatory cytokines synergistically, keratinocytes are highly proliferative and can produce copious chemokines (e.g. CXCL1/2/3, CXCL8, CXCL9/10/11, CCL2, and CCL20) to recruit leukocytes (such as neutrophils, Th17 cells, dendritic cells, and macrophages), antimicrobial peptides (e.g. S100A7/8/9/12, hBD2, and LL37) to induce innate immunity, and other inflammatory mediators to amplify inflammation. Moreover, keratinocytes, together with fibroblasts and endothelial cells, lead to tissue reorganization via activation and proliferation of endothelial cells and deposition of extracellular matrix [8–10]. The crosstalk between keratinocytes and immune cells especially Th17 cells results in the induction and maintenance of psoriasis with hyperproliferation and aberrant differentiation of keratinocytes, dilated and hyperplastic blood vessels, and infiltration of inflammatory cells like leukocytes [7, 11, 12].

## Factors regulating psoriatic keratinocytes

### Genetic regulation

Epidemiologic studies indicate a strong genetic basis for psoriasis with a high heritability rate of 60–90% [13]. Over 80 psoriasis-susceptible loci have been identified, the strongest of which was the psoriasis-associated susceptibility locus 1 (PSORS1). Throughout these decades, there are at least 100 susceptibility genes for psoriasis identified, most of which are involved in adaptive immunity, innate immunity, and skin barrier function [13, 14]. Some of the candidate causal genes are keratinocyte-associated, which will be discussed below and summarized in Table 1.

**Table 1:** Characteristics of mutations of gene associated with keratinocytes in psoriasis.

Gene name	Chromosomal locus	Mutation	Protein function	Ref.
CARD14	17q25.3	Gain-of-function mutations	Activation of NF-κB signaling	[6, 15–18]
TNFAIP3	6q23.3	Loss-of-function mutations	Inhibition of NF-κB signaling	[19]
TNIP1	5q33.1	Loss-of-function mutations	Inhibition of NF-κB signaling	[20, 21]
VEGFA	6p21.1	Controversial	A main proangiogenic factor	[22–27]
TRAF3IP2	6q21	Loss-of-function mutations	Signal transduction downstream of IL-17A	[28, 29]
IFI27	14q32.12	N/A	Epidermal growth factor-stabilized protein	[30, 31]
IL-36RN	2q14.1	Reduced-/loss-of-function mutations	Inhibition of NF-κB signaling	[32–34]
AP1S3	2q36.1	Loss-of-function mutations	Involved in autophagy	[35]

*CARD14* caspase recruitment domain family member 14, *TNFAIP3* TNF-α induced protein 3, *TNIP1* TNFAIP3-interacting protein 1, *VEGFA* vascular endothelial growth factor A, *TRAF3IP2* TRAF3 interacting protein 2, *IFI27* interferon alpha-inducible protein 27, *IL-36RN* IL-36 receptor antagonist, *AP1S3* adaptor related protein complex 1 subunit sigma 3, *N/A* not available.

Genomic studies linked the mediators of NF-κB pathway as disease susceptibility genes for psoriasis. For example, CARD14, encoding scaffold protein CARMA2 (CARD-containing MAGUK protein 2), is a NF-κB activator, which is mainly expressed in epidermal keratinocytes. Several genetic mutations of CARD14 (which maps to the psoriasis susceptibility locus 2 (PSORS2)) have been identified associated with psoriasis susceptibility [6]. To investigate the pathogenic role of CARD14 mutation in psoriasis, two groups generated two mouse models with patient-derived CARD14 gain-of-function mutations (a mutation in E138 (Card14^E138A/+^) or a deletion of E138 (Card14^∆E138A/+^)), respectively. Spontaneous psoriasis-like phenotype was developed in both models, indicating the CARD14 gain-of-function mutation is sufficient to drive the initiation of psoriasis [15, 16]. Furthermore, CARD14 deficiency protected mice from IMQ- or IL-23-induced psoriasis-like dermatitis [15, 17]. Mechanically, gain-of-function CARD14 mutations caused CARD14 aggregation and hyperactivation and enhanced CARD14-BCL10-MALT1 complex formation in keratinocytes, which results in constitutive activation of NF-κB and MAPK signaling pathway, subsequently leading to elevated expression of inflammatory cytokines, chemokines, and antimicrobial peptides and enhanced activation of IL23/IL-17A axis [15, 16, 18]. Of note, CARD14 serves as a key mediator in IL-23/IL-17A axis through interaction with ACT1-TRAP6 signaling complex [15].

In addition, the NF-κB inhibitors TNFAIP3 (TNF-α induced protein 3, encoding A20) and TNIP1 (TNFAIP3-interacting protein 1, encoding ABIN1) have been identified as susceptibility loci for psoriasis and keratinocyte-associated genes. TNFAIP3/A20 is a deubiquitinase that can be linked to the IkB kinase complex by TNIP1/ABIN1, thus preventing activation of NF-κB. TNFAIP3/A20 was decreased in the epidermis of psoriatic patients, and keratinocyte-specific deletion of TNFAIP3/A20 potentiates the pro-inflammatory genes expression of keratinocytes in psoriasis and other inflammatory disorders [19]. Global deletion of TNIP1/ABIN1 caused mice susceptible to the development psoriasis-like dermatitis induced by IMQ. Further investigation indicated TNIP1/ABIN1 deficiency in keratinocytes was sufficient to promote psoriasis inflammation, which disturbed IL-17-induced gene expression, and exaggerated chemokine and cytokine production [20]. Furthermore, the inhibitory effect of TNIP1/ABIN1 on psoriasis is mainly attributed to the suppression of inflammatory responses in keratinocytes rather than inhibiting keratinocyte proliferation [21].

Vascular endothelial growth factor A (VEGFA), the main epidermal-derived vessel-specific growth factor, is overexpressed in several inflammatory diseases, including psoriasis. The VEGFA gene is located at the PSORS1 locus, which is highly polymorphic and associated with psoriasis severity [22]. Transgenic mouse model overexpressing VEGFA in keratinocytes developed a spontaneous psoriasiform phenotype and inhibition of VEGFA reversed the psoriatic phenotype mediated by epidermal overexpression of VEGFA or epidermal deletion of c-Jun/JunB [23–26]. VEGFA works through its receptors Flt1 (VEGFR1) and Flk1 (VEGFR2) and its coreceptor Neuropilin 1 (Nrp1). Keratinocyte-specific deletion of Flt1 or Nrp1 diminished VEGFA-induced psoriasis, suggesting that VEGFA/Flt1/Nrp1 axis has an essential epidermal autonomous function in the pathogenesis of psoriasis [27]. Altogether, these results indicate the epidermal VEGFA signaling as a promising therapeutic target for psoriasis.

TRAF3 interacting protein 2 (TRAF3IP2) is another psoriasis susceptibility gene associated with keratinocytes. It encodes ACT1, which is an adaptor protein with ubiquitin ligase activity, and plays an essential role in the signal transduction downstream of IL-17A receptor. TRAF3IP2 silencing in keratinocytes enhanced cell differentiation and inhibited IL-17 response [28]. In vivo, loss of ACT1 inhibited IL-17 signaling pathway and protected mice from psoriasiform dermatitis induced by IMQ and IL-23 [28, 29]. Interferon alpha-inducible protein 27 (IFI27) maps chromosome 14q32, which is located at a psoriasis susceptibility locus [30]. IFI27 was upregulated in the lesional skin of psoriatic patients and serves as a novel epidermal growth factors-stabilized protein in keratinocytes. Silencing IFI27 in keratinocytes caused cell cycle arrest and inhibited cell proliferation, and topical application of IFI27 siRNA ameliorated IMQ-induced epidermal hyperplasia in mice [31].

Except for mutations in CARD14, mutations in IL-36 receptor antagonist (IL-36RN) and adaptor-related protein complex 1 subunit sigma 3 (AP1S3) have also been identified to cause or contribute to pustular psoriasis that is a rare and severe form of psoriasis. IL-36Ra (encoded by IL-36RN) is released by keratinocytes and accumulates in the initial phase of psoriasis after inflammatory cytokines stimulation [32]. Loss of IL-36Ra in mice exacerbated IMQ-induced psoriasis-like dermatitis [33]. Mutations in IL36RN results in a reduced/loss of function of IL-36Ra and therefore enhances IL-36 and NF- κB signaling in keratinocytes [34]. AP1S3 is highly expressed in keratinocytes, with low expression in neutrophils or undetectable expression in CD4^+^ T cells. Mutations of AP1S3, such as p.Phe4Cys and p.Arg33Trp, are loss-of-function mutations and AP1S3 deficiency led to autoinflammation mediated by impaired keratinocyte autophagy and increased IL-36 signaling, finally contributing to the development of psoriasis [35].

### Cytokines and receptors

**Fig. 2:**
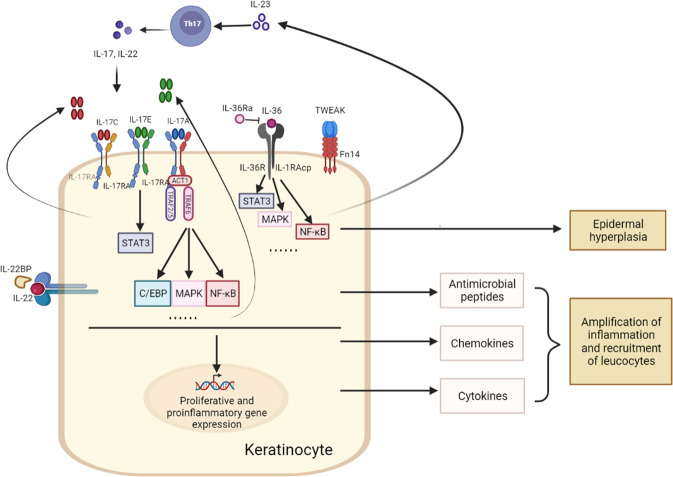
The role of cytokines derived from or receptors expressed on keratinocytes in psoriasis. Cytokines are essential in the pathogenesis of psoriasis. Recently, cytokines derived from or receptors expressed on keratinocytes have shown great importance in psoriasis. Keratinocytes are critical cytokine responders in psoriasis, as keratinocyte-specific deletion of their receptors (such as IL-17RA and IL-36R) alleviated psoriasiform lesion in psoriatic mouse model. Keratinocyte-derived IL-17C, IL-17E, IL-36, and IL-23 could induce expression of proliferative and proinflammatory genes by multiple signaling pathways, leading to epidermal hyperplasia and amplification of inflammation and leukocyte infiltration. IL-17RA IL-17 receptor A, TRAF6 TNF receptor-associated factor 6, IL-36R IL-36 receptor, IL-1RAcp IL-1 receptor accessory protein, TWEAK tumor necrosis factor (TNF)-like weak inducer of apoptosis, Fn14 factor-inducible 14, IL-22BP IL-22 binding protein.

Communication between immune cells and keratinocytes is through cytokines and their receptors, which plays a pivotal role in psoriasis pathogenesis. Mainly produced by immune cells, TNF-α, IFN-γ, IL-23/IL-17A, IL-22, etc, activate keratinocytes, triggering multiple cell signaling pathways, ultimately resulting in excessive keratinocytes proliferation and production of antimicrobial proteins, cytokines, chemokines, and growth factors. Among them, TNF-α, IL-17A, and IL-23 are of central importance in psoriasis as therapies targeting them are most efficient in the treatment of patients [10]. Recently, cytokines derived from or receptors expressed on keratinocytes have attracted more attention from researchers (Fig. 2).

The IL-23/IL-17 cytokine axis is considered as a major driver of psoriasis. IL-23 expressed by immune cells is believed to be required for the maintenance and expansion of IL-17-producing immune cells [10]. However, IL-23 is also produced by keratinocytes, but the role of keratinocyte-produced IL-23 in psoriasis is unclear. Recently, using a genetic mouse model, Li and colleagues showed that keratinocyte-derived IL-23 was sufficient to activate IL-17-producing immune cells to secrete IL-17 and cause a chronic skin inflammation. Further investigation found that epigenetic regulation by H3K9 dimethylation controled IL-23 expression in keratinocytes, which may contribute to psoriasis [36].

The IL-17 family cytokine contains six members: IL-17A (commonly referred to as IL-17), IL-17B, IL-17C, IL-17D, IL-17E (IL-25), and IL-17F, which acts through a IL-17 receptor heterodimer. IL-17A, the major downstream cytokine of IL-23, is most strongly implicated and well-studied in psoriasis pathogenesis [37]. Briefly, IL-17A binds to its receptors on keratinocytes, through multiple cell signaling pathways, it induces the production of keratinocyte-derived antimicrobial peptides (e.g. S100A7, LL37, and DEFB4A) to activate innate immunity, chemokines (e.g. CXCL1, CXCL8, and CCL20) to recruit leukocytes such as neutrophils, Th17 cells, mDCs and macrophage, and multiple pro-inflammatory genes (such as IL-1β, IL-6, IL-8, and TNF-α), thus amplifying the IL-23/IL-17A axis and producing the “feed forward” inflammatory circuits. On the other hand, IL-17A could indirectly induce epidermal hyperplasia via increased expression of IL-19 and IL-36 by keratinocytes [11, 37]. However, there has been less attention on other IL-17 family members, despite of their upregulation in psoriatic lesions. Like IL-23, IL-17E (IL-25) is derived from both immune cells and keratinocytes, which was highly expressed in the epidermal layer of lesional skin of psoriatic patients and IMQ-induced psoriasis mouse model. IL-17E injection caused psoriasis-like pathology in mouse skin, whereas global knockout of IL-17E ameliorated IMQ-induced psoriasis. Specially, IL-17E deficiency in keratinocytes could lead to resistance to IMQ-induced psoriasis. Mechanically, epidermal IL-17E expression is upregulated by IL-17A in psoriasis, and through its receptor IL-17RB on keratinocytes, it promotes keratinocyte proliferation and production of inflammatory cytokines and chemokines via STAT3 activation [38]. IL-17C is another IL-17 family member that has been identified as an epithelial cytokine predominantly produced by keratinocytes in skin [38]. IL-17C is reported to be increased in keratinocytes in a number of inflammatory skin diseases, such as psoriasis and atopic dermatitis (AD) [39]. And keratinocyte-specific IL-17C transgenic mice developed a spontaneous psoriasis-like phenotype [40]. Using MOR106, a specific anti-IL-17C antibody, it attenuated keratinocyte hyperproliferation and skin inflammation in mouse models of psoriasis and AD [41]. Of note, IL-17C builds a self-amplifying circuit, which results in enhanced production of inflammatory cytokines, chemokines, and antimicrobial peptides by keratinocytes, ultimately recruiting immune cells to the skin. It is noteworthy that IL-17C is not a specific target for psoriasis and AD but for a variety of inflammatory skin diseases.

The IL-17 receptor family contains five members, including IL-17RA, IL-17RB, IL-17RC, IL-17RD, and IL-17RE. IL-17RA is the most common co-receptor subunit of IL-17A, IL-17C, IL-17E, and IL-17F. Recently, Moos and colleagues determined the critical cell type responding to IL-17 by using different murine models with IL-17RA deficient in distinct cell types, such as keratinocytes, T cells, neutrophils, and macrophages. They found that only IL-17RA deletion in keratinocytes significantly protected mice from IMQ-induced psoriasis-like dermatitis, which is similar to full-body deficiency of IL-17RA. Importantly, mice lacking IL-17RA in keratinocytes also featured significantly decreased expression of IL-1, IL-22, and CXCL2, as well as loss of neutrophil infiltration into the skin. However, deletion of IL-17RA in T cells, neutrophils or macrophages showed no impact on disease development [42]. Thus, keratinocytes are the crucial IL-17 responder in psoriasis.

The IL-36 family is a member of IL-1 superfamily and comprises three agonists (IL-36α, IL-36β, and IL-36γ) and one antagonist (IL-36 receptor antagonist (IL-36Ra)). All of the members bind to a heterodimeric receptor complexes composed of IL-36 receptor (IL-36R) and IL-1 receptor accessory protein (IL-1RAcp). Now, the IL-36 family cytokines are emerging as crucial players in the pathogenesis of psoriasis. Loss of IL-36Ra exacerbated IMQ-induced psoriasiform lesion [33]. By contrast, mice with IL-36α deficiency (but not IL-36β or IL-36γ deficiency), had significantly reduced psoriasis-like phenotype induced by IMQ [33, 43]. Similarly, IL-36R deficiency protected mice from IMQ-induced psoriasis-like dermatitis [33]. Specially, conditional deletion of IL-36R in keratinocytes showed similar protection as global deficiency of IL-36R [44], suggesting keratinocyte is the primarily responsive cell type for IL-36 signaling in psoriasis. Notably, IL-36 exerts its pathogenic role by promoting keratinocyte proliferation and enhancing the production of inflammatory cytokines and chemokines to amplify psoriatic inflammation [45–51]. Recently, therapies targeting IL-36 have been developed for psoriasis treatment, which are under clinical trials.

IL-22 is another major downstream cytokine of IL-23, which is mainly produced by CD4^+^ T cells and group 3 innate lymphoid cells (ILC3). Its receptor IL-22R is expressed on non-hematopoietic cells such as keratinocytes, epithelial cells, and hepatocytes [52]. IL-22 exerts its pathogenic role in psoriasis by inhibiting the terminal differentiation of keratinocytes, as well as inducing antimicrobial peptides and proinflammatory chemokines [53]. IL-22 binding protein (IL-22BP) is a natural inhibitor of IL-22 that specially binds to IL-22, thereby inhibiting its biological function. Both genetic IL-22BP deficiency and anti-IL-22BP neutralizing antibody exacerbated IMQ-induced psoriasis-like skin disease with increased levels of epidermal thickeness and enhanced expression of inflammatory cytokines and IL-22-inducible antimicrobial peptides. Further investigation found that IL-24 is a downstream target of IL-22, regulating the terminal differentiation of keratinocytes [54].

Except for TNF-α, another member of TNF superfamily, TNF-like weak inducer of apoptosis (TWEAK) has recently been identified as a key cytokine in psoriasis. Daniel and colleagues showed that TWEAK deficiency alleviated IMQ-induced psoriatic dermatitis [55]. In addition, mice deficient in fibroblast growth factor-inducible 14 (Fn14, the receptor for TWEAK) also reduced the disease severity [56]. Most recently, using a mouse model with keratinocyte-specific deletion of Fn14, Rinkesh and colleagues have demonstrated a central role of keratinocytes in the action of TWEAK in psoriasis. Loss of Fn14 in keratinocytes protected mice from IMQ-induced psoriasiform hyperplasia and inflammation. Importantly, blocking TWEAK showed a similar reduction in epidermal thickness, skin infiltrates, and inflammation mediators as blocking TNF-α and IL-17A. However, there was no further improvement with combined treatments [57]. Altogether, blocking TWEAK may be an alternative therapeutic strategy for psoriasis.

### Metabolic mechanism

One of the hallmark of psoriasis is keratinocyte hyperproliferation, which requires extensive energy, amino acids, nucleotides and lipids. In recent years, emerging evidence has implicated that metabolism is essential in the pathogenesis of psoriasis, especially in keratinocytes.

Glucose is the main source of energy for all cells, particularly for rapidly proliferating cells. Now, glucose metabolism is being recognized as a key metabolic mechanism involved in psoriasis. Glucose uptake is through glucose transporters, and glucose transporter 1(Glut1) is most widely expressed and dramatically elevated in psoriatic epidermis from the patient and IMQ-induced psoriasis mouse model [58, 59]. Keratinocyte-specific deletion of Glut1 did not affect normal skin development and homeostasis, but ameliorated IMQ- and IL-23-induced psoriasiform hyperplasia. Furthermore, topical application of GLUT inhibitor WZB117 attenuated both posriasiform hyperplasia and inflammation in mouse models of psoriasis, identifying glucose transport as a promising therapeutic target of psoriasis [59]. Also, suppressing glucose metabolism by 2-deoxy-d-glucose (2DG), a glucose analog, inhibited keratinocyte proliferation and alleviated IMQ-induced skin lesions [58, 60, 61]. Glycolysis and aerobic respiration are also related with keratinocyte function in psoriasis. Recently, a key rate-limiting enzyme of glycolysis, pyruvate kinase M2 (PKM2), was found significantly increased in the lesional skin of psoriatic patients and IMQ-induced psoriasis-like dermatitis. Overexpression of PKM2 increased keratinocyte glucose metabolism, whereas silencing or inhibition of PKM2 suppressed keratinocyte cell glycolysis and proliferation. Moreover, genetic deletion of PKM2 in keratinocytes or pharmacological inhibition of PKM2 markedly reduced psoriasis-like skin lesions induced by IMQ. Importantly, EGF may contribute to the induction of PKM2 in keratinocyte through ERK1/2 pathway [61].

In addition to glucose metabolism, glutamate metabolism has been reported to be abnormal in psoriasis. Multiple metabolomics analysis revealed an elevation of glutamate metabolism in patients with psoriasis, which is positively correlated with the Psoriasis Area Severity Index (PASI) score. Glutamate metabolism serves a crucial role in psoriasis, as it may facilitate the hyperproliferating keratinocytes to meet their high metabolic demand, such as ATP or biosynthetic procurers [62–65]. Recently, Xia and colleagues found that Glutaminase 1(GLS1)-mediated glutaminolysis was aberrantly activated in psoriasis patients, as indicated by elevated mRNA and protein levels in both immune cells and keratinocytes, contributing to the pathogenesis of psoriasis. In keratinocytes, induction of GLS1 was caused by IL-17A/MALT1/c-Jun axis, and enhanced cell proliferation and chemokine production, contributing to the development of psoriasis phenotype [66].

Emerging evidence also suggests the importance of lipid metabolism in psoriasis. Studies analyzing plasma or serum showed altered lipid metabolites in psoriatic patients [67, 68]. In psoriatic skin lesions, lipid metabolism abnormalities were also observed [69, 70]. And our high-throughput transcriptome analysis of psoriatic skins identified notable differences in genes involved in lipid metabolism [71]. Sphingosine-1-phosphate (S1P), a metabolic product of sphingolipids, has been reported to be elevated in patients with psoriasis. It is a bioactivator that acts both as an intracellular second messenger and an extracellular ligand for G-protein-coupled receptors, which is involved in diverse cellular processes, including immune cell trafficking and keratinocyte proliferation and differentiation [72, 73]. To date, the role of S1P in psoriasis is controversial. Schaper and colleagues found that topical application of S1P alleviated IMQ-induced epidermal thickening and skin inflammation in the ear [74]. And using a selective S1P1 receptor agonist- Sy1930, it attenuated propranolol-induced psoriasis in pigs [75]. Furthermore, Jeon and colleagues showed that elevating S1P by inhibiting of S1P lyase ameliorated IMQ-induced psoriasis-like dermatitis, and reduced IL-17- and IL-22-induced cell proliferation and promoted keratinocyte differentiation [76]. However, blocking S1P generation by ceramidase inhibitor or sphingosine kinase 1/2 inhibitor protected mice from IMQ- induced skin lesions and inflammation, especially through inhibiting Th17 cell differentiation. Thus, it would be better to use conditional knockout mice to study the role of S1P in psoriasis, to dissect its role in immune cells and keratinocytes. Proprotein convertase subtilisin/kexin type 9 (PCSK9) is a chaperone protein to the low-density lipoprotein (LDL) receptors, which promotes the degradation of LDL receptors. Induction of PCSK9 was observed in the lesional skin of both psoriatic patients and IMQ-induced psoriasis mouse model. Genetic deletion of PCSK9 or topical application of siRNA targeting PCSK9 relieved the psoriasis-like inflammation as well as the proliferation of keratinocytes. Notably, silencing PCSK9 in keratinocytes induced cell apoptosis and inhibited hyperproliferation [77].

Cellular metabolism in keratinocytes not only affects the supply of energy for keratinocyte proliferation but is also involved in a variety of inflammatory and immune response.

### Cell signaling

The signaling pathway is a major regulator of psoriasis involved in diverse biological aspects we discussed in this review, which influences both immune cells and keratinocytes. Major signaling pathways altered in psoriasis include signal transducer and activator of transcription (STAT), nuclear factor-kappa B (NF-κB), MAPK, etc. In this section, only the key regulators of major signaling pathways in keratinocytes will be discussed and other factors involved in cell signaling may be discussed in other sections.

#### STAT signaling pathways

The JAK (Janus kinase)/STAT signaling is known to play an essential role in psoriasis. Of note, among the various STATs, STAT3 is hyperactivated in both immune cells and keratinocytes, regulating cell proliferation, differentiation, and apoptosis. In keratinocytes, STAT3 has a central role in response to various inflammatory cytokines in psoriasis, such as IL-6, IL-17, IL-21, IL-19, IL-22, etc. STAT3 activation in keratinocytes inhibited cell differentiation, promoted proliferation and production of antimicrobial peptides [78, 79]. The transgenic mouse model overexpressing STAT3 in keratinocytes led to the spontaneous development of psoriasis-like lesions with similar cytokine profiles as those of human psoriatic plaques [80, 81]. Moreover, specific deletion of STAT3 in keratinocytes rather than in T cells reduced psoriasis-like dermatitis [82]. Thus, STAT3 in keratinocytes is more important for the development of psoriasis.

#### NF-κB signaling pathway

Increasing evidence has shown that NF-κB signaling contributes to the pathogenesis of psoriasis by acting on immune cells and keratinocytes. NF-κB was highly activated in the lesional skin of psoriatic patients [83]. Using different mouse models, Bernd and colleagues showed that aberrant activation of NF-κB in both keratinocytes and T cells are important for the development of inflammatory skin diseases, like psoriasis. Mice with global deletion of IκBα developed psoriasis-like skin symptoms, while IκBα deficiency in keratinocytes only resulted in epidermal hyperplasia without epidermal inflammation. However, loss of IκBα in both keratinocytes and T cells led to a similar phenotype as that in global deficiency. Moreover, keratinocyte-specific deletion of RelA rescued the phenotype developed in global IκBα knockout mice [84]. And mice deficient in NF-κB alleviated IMQ-induced psoriasis-like dermatitis [85]. These indicate NF-κB activation in both keratinocytes and immune cells are essential for the development of psoriasis.

#### MAPK signaling pathway

MAPK kinases are involved in the pathogenesis of psoriasis, and play important roles in regulating keratinocyte proliferation and immune response. p38 was activated in psoriatic epidermis and cutaneous activation of p38 resulted in psoriasis-like dermatitis in mice. Topical application of p38 inhibitor attenuated IMQ-induced dermatitis [86]. In vitro studies showed that p38 inhibitor suppressed TNF-α or IL-17A-stimulated inflammatory response in keratinocytes [86, 87]. Like p38, ERK1/2 was also activated in the epidermis of psoriatic patients [88]. Inhibition of ERK by a specific ERK inhibitor JSI287 decreased IMQ-induced psoriasiform lesion [89]. DUSP1/MKP-1, a member of the dual-specificity phosphatase family, acts as a negative regulator of MAPK pathway. It was significantly downregulated in psoriasis patients and overexpression of DUSP1 markedly inhibited keratinocyte proliferation and promoted apoptosis by targeting ERK/Elk-1/Egr-1 signaling pathway [90].

#### Other signaling pathways

Secreted frizzled-related protein (SFRP) 4, a negative regulator of Wnt, is pivotal for epidermal hyperplasia. SFRP4 was decreased in the skin epidermis of psoriatic patients and mouse models of psoriasis by an epigenetic regulation- DNA methylation. SFRP4 treatment or Wnt inhibition suppressed keratinocyte hyperproliferation induced by IL-6 in vitro. Administration of SFRP4 or pharmacological inhibition of Wnt alleviated psoriasis-like dermatitis induced by IMQ [91]. Recently, Hippo-Yes-associated protein (YAP) signaling has been reported to be involved in psoriasis. YAP and its downstream target amphiregulin (AREG) were dramatically induced in skin of psoriatic patients and in psoriasis-like mouse model. As an oncogene, YAP promotes cell proliferation and inhibits cell apoptosis. And silencing YAP inhibited keratinocyte proliferation, induced cell cycle arrest, and promoted cell apoptosis, which acts through an AREG-dependent pathway [92].

### Transcription factor

Except for the transcription factors involved in the major signaling pathways in psoriasis discussed above, some other transcription factors expressed in keratinocytes have emerged as important regulators in psoriasis, which have appealing therapeutic potential.

Nuclear factor erythroid 2-related factor 2 (Nrf2) is a transcription factor that plays a critical role in regulating cellular defenses against oxidative or toxic stresses. In psoriasis, Nrf2 was highly expressed and activated in psoriatic epidermis. Overexpression of Nrf2 facilitated keratinocyte proliferation by increasing the expression of hyperproliferation-associated keratins, including keratin (KRT) 6, KRT16, and KRT17. Locally silencing Nrf2 reduced IMQ-induced psoriasis-like dermatitis and inhibited KRT6, KRT16, and KRT17 expression [93]. These indicate Nrf2 acts as a regulator of hyperproliferation-associated keratins, which is a potential therapeutic target for psoriasis. AP-1 transcription factor superfamily (including JUN, JUNB, JUND, FOS, FOSB, FRA1, and FRA2) is essential in the pathogenesis of psoriasis. Epidermal deletion of c-Jun and JunB led to a spontaneous psoriasiform phenotype [94]. FRA1 was evidently elevated in the lesional skin of psoriatic patients. Overexpression of FRA1 in keratinocytes triggerred enhanced production of proinflammatory cytokine and chemokine and promoted keratinocyte migration and wound healing process [95]. Grainyhead-like 3 (GRHL3) is a transcription factor that plays a critical role in epidermal differentiation and barrier formation. Upregulation of GRHL3 was observed in the epidermis of both psoriatic patients and IMQ-induced psoriasis mouse model. GRHL3 deficiency in keratinocytes aggravated IMQ-induced psoriasiform phenotype [96]. Further investigation identified thymus and activation-regulated chemokine (TARC) as a downstream of GRHL3 that promoted keratinocyte proliferation after loss of GRHL3 [97].

### Non-coding RNAs

Besides genetic factors, epigenetic regulation is also involved in psoriasis. Non-coding RNA regulation is one of the epigenetic regulations important for various biological processes and disease pathogenesis. The role of non-coding RNAs in psoriasis, especially the role of microRNAs has been extensively explored previously [98, 99]. Here, we will focus on the effects of non-coding RNAs (microRNA and long non-coding RNA (lncRNA)) on keratinocytes in psoriasis.

#### Role of microRNAs in psoriatic keratinocytes

MicroRNAs are short non-coding RNAs, which inhibit protein-coding gene expression at the post-transcriptional level and their dysfunction is linked to psoriasis [100]. Early studies observed that more than 250 microRNAs were aberrantly expressed in the lesional skin of psoriatic patients, and play an essential role in modulating the functions of multiple cell types important for psoriasis pathogenesis, such as keratinocytes and leukocytes, as well as the interplay between them [98, 101]. Here, we will discuss how microRNAs affect psoriatic keratinocytes and psoriasis, and the summary of microRNAs involved in keratinocytes is shown in Table 2.

**Table 2:** Characteristics of microRNAs regulating keratinocytes in psoriasis.

miRNA	Level	Site of found	Model	Target gene	Effects on psoriasis	Ref.
miR-17-92	Upregulated	Lesional of psoriatic patients	Cytokine cocktail-treated NHKs	CDKN2B, SOCS1	Pathogenic, promote cell-cycle progression, keratinocyte proliferation, and chemokine production	[103]
miR-31	Upregulated	Lesional skin of psoriatic patients and IMQ‐treated mouse models	IL-6-treated NHEK and HaCaT cells; IMQ‐treated mouse	PPP6c	Pathogenic, promote cell-cycle progression, and keratinocyte proliferation	[102]
miR-130a	Upregulated	Lesional skin of psoriatic patients	HaCaT	STK40	Pathogenic, promote cell viability and migration, and inhibit apoptosis of keratinocytes	[104]
miR-146a/b	Upregulated	Lesional skin of psoriatic patients	IFN-γ or TNF-α-treated NHEK & IMQ‐treated mouse	TRAF6, EGFR, NUMB, FERMT1	Protective, inhibit keratinocyte proliferation, and inflammatory responses	[100, 106–108]
miR-223	Upregulated	Lesional skin and PBMCs of psoriatic patients	IL-22-treatedHaCaT, HEK293	PTEN	Pathogenic, promote proliferation, and inhibits apoptosis of keratinocytes	[105]
miR-20a-3p	Downregulated	Lesional skin of psoriatic patients	IL-22-treated HaCaT	SFMBT1	Protective, inhibit proliferation, and promote apoptosis of keratinocytes	[117]
miR-125b (miR-125b-5p)	Downregulated	Lesional skin of psoriatic patients	HEKs	FGFR2, USP2, AKT3	Protective, inhibit keratinocyte proliferation	[114, 115]
miR-138	Downregulated	Lesional skin of psoriatic patients	HaCaT	hTERT	Protective, inhibits proliferation, and promotes apoptosis of keratinocytes	[113]
miR-145-5p	Downregulated	Lesional skin of psoriatic patients	IL-17A-treated-NHEK; IMQ-treated mouse	MLK3	Protective, inhibit keratinocyte proliferation, and chemokine production	[110]
miR-181b (miR-181b-5p)	Downregulated	Lesional skin of psoriatic patients	HEKs	TLR4, AKT3	Protective, inhibit keratinocyte proliferation	[114, 116]
miR-187	Downregulated	Lesional skin of psoriatic patients	IL-6-treated-HaCaT; IMQ‐treated mouse	CD276	Protective, inhibit keratinocyte proliferation	[111]
miR-217	Downregulated	Lesional skin of psoriatic patients	HEKn	GRHL2	Protective, inhibit proliferation, and promote differentiation of keratinocytes	[120]
miR-330	Downregulated	Lesional skin of psoriatic patients	IL-22-treated HaCaT & HKC	CTNNB1	Protective, inhibit keratinocyte proliferation	[118]
miR-486-3p	Downregulated	Lesional skin of psoriatic patients	HaCaT, TNF-α, IL-17, IL-22-treated NHEK	K17	Protective, inhibit keratinocyte proliferation	[112]
miR-876-5p	Downregulated	Lesional skin and blood of psoriatic patients	HaCaT	Ang-1	Protective, inhibits proliferation, invasion, and adhesion of epidermal keratinocytes	[119]
miR-let-7b	Downregulated	Psoriatic epidermis of IMQ-treated mouse models	HaCaT; IMQ‐treated mouse	IL-6	Protective, promote keratinocyte differentiation	[109]

*miR* microRNA, *NHKs* normal human keratinocytes, *CDKN2B* cyclin-dependent kinase inhibitor 2B, *SOCS1* suppressor of cytokine signaling 1, *IMQ* imiquimod, *IL-6* interleukin-6, *NHEK* normal human epidermal keratinocytes; *HaCaT* human-immortalized epidermal cell line, *PPP6c* protein phosphatase 6, *STK40* specific serine/threonine kinase 40, *IFN-γ* interferon γ, *TNF-α* tumor necrosis factor α, *TRAF6* TNF receptor–associated factor 6, *EGFR* epidermal growth factor receptor, *FERMT1* fermitin family member 1, *PBMCs* peripheral blood mononuclear cells, *HEK293* human embryo kidney 293, *PTEN* phosphatase and tensin homolog deleted on chromosome ten, *SFMBT1* Scm like with four mbt domains 1, *HEKs* human epidermal keratinocytes, *FGFR2* fibroblast growth factor receptor 2, *USP2* ubiquitin-specific peptidase 2, *AKT3* AKT serine/threonine kinase 3, *hTERT* human telomerase reverse transcriptase, *MLK3* mixed-lineage kinase 3, *TLR4* toll-like receptor 4, *CD276* cluster of differentiation 276, *HEKn* human epidermal keratinocytes, neonatal, *GRHL2* grainyhead-like 2, *HKC* human renal tubular epithelial cell, *CTNNB1* catenin beta 1, *K17* keratin 17, *Ang-1* Angiopoietin-1.

Some microRNAs show aberrant upregulation in psoriasis and mainly promote keratinocyte proliferation. For example, microRNA (miR)-31 is upregulated in psoriatic skin of patients or IMQ-induced mouse model, especially in the basal or superbasal cell layers of epidermis. Genetic deletion of miR-31 in keratinocytes alleviated psoriasiform hyperplasia and inflammation induced by IMQ or IL-23. Of note, miR-31 was induced by NF-κB, which in turn suppressed protein phosphatase 6 (ppp6c), and thereby promoted the G1 to S phase progression, finally resulting in increased proliferation of keratinocytes in psoriasis [102]. miR-17-92 cluster, including miR-17, miR-18a, miR-19a, miR-19b miR-20a, and miR-92a, was also induced in psoriatic lesions and positively correlated with PASI. Zhang and colleagues found that STAT1-induced miR-17-92 cluster promoted keratinocyte proliferation by suppressing cyclin-dependent kinase inhibitor 2B (CDKN2B) and increased the chemokine production via inhibition of suppressor of cytokine signaling 1 (SOCS1), suggesting miR-17-92 as a potential therapeutic target for psoriasis [103]. Moreover, miR-130a, highly expressed in psoriatic lesions, directly targeted serine/threonine kinase 40 (STK40) to activate NF-κB pathway or indirectly upregulated sex-determining region Y chromosome-box 9 (SOX9) to activate JNK/MAPK pathway, thus promoting keratinocytes viability and migration and inhibiting apoptosis of keratinocytes [104]. In addition, miR-223 targeted phosphatase and tensin homolog (PTEN), a tumor suppressor inhibiting the PI3K/AKT signaling pathway, and ultimately contributed to increased proliferation and decreased apoptosis in IL-22-stimulated HaCaT cells [105]. However, miR-146a/b, which were highly expressed in the psoriatic skin lesions, negatively regulated keratinocyte proliferation and played protective roles in psoriasis. miR-146a targeted TNF receptor-associated factor 6 (TRAF6) and epidermal growth factor receptor (EGFR), which regulated keratinocyte proliferation and inflammatory responses [106, 107]. Overexpression of miR-146a inhibited epidermal proliferation, impaired neutrophil infiltration, and suppressed IL-17-driven psoriatic inflammation of mouse models via its target genes, while genetic deficiency in miR-146a exacerbated pathology of psoriasis-like skin inflammation especially in the early onset of the disease [101]. miR-146b facilitated miRNA-146a to inhibit the proliferation and psoriasis-related target gene expression (such as FERM domain containing kindlin 1 (FERMT1), NUMB endocytic adaptor protein (NUMB), etc.) in cultured human keratinocytes stimulated with IFN-γ or TNF-α [108].

Besides, some microRNAs are aberrantly downregulated in psoriatic skin lesions and exert proliferation-inhibiting and differentiation-promoting effects on keratinocytes. miR-let-7b, the first known human microRNA, was dramatically reduced in psoriatic epidermis of IMQ-induced psoriasis mouse model. Using a keratinocyte-specific miR-let-7b transgenic mouse model, Wu and colleagues showed that overexpression of miR-let-7b in keratinocytes ameliorated psoriasis-like dermatitis induced by IMQ. Mechanically, miR-let-7b inhibited ERK signaling pathway through targeting IL-6, resulting in the acceleration of keratinocyte differentiation in psoriasis [109]. Downregulation of miR-145-5p was observed in the lesional skin of psoriatic patients. Overexpression of miR-145-5p inhibited cell proliferation and chemokine production by targeting mixed-lineage kinase 3 (MLK3)-mediated NF-κB and STAT3 activation in vitro and alleviated psoriasiform hyperplasia and inflammation in vivo. By contrast, inhibition of miR-145-5p led to the opposite effects [110]. Similarly, overexpression of miR-187 suppressed keratinocyte hyperproliferation and protected mice from IMQ-induced skin lesions through inhibition of CD276-STAT3 signaling [111]. miR-486-3p was markedly decreased in the epidermis of psoriatic patients and showed a negative correlation with the disease severity. TGFβ/SMAD was identified as an upstream of miR-486-3p. In psoriasis, inactivation of TGFβ/SMAD pathway led to the loss of miR-486-3p, resulting in KRT17 (a cytoskeletal protein that play a pathogenic role in psoriasis) overexpression and keratinocyte hyperproliferation [112]. In line with miR-486-3p, miR-138 is another negative regulator of KRT17 by targeting human telomerase reverse transcriptase (hTERT), subsequently inhibiting keratinocyte proliferation and increasing cell apoptosis [113]. Moreover, miR-125b-5p and miR-181b-5p shared the same target gene AKT3, which at least partly contributed to the inhibition of keratinocyte proliferation in psoriasis [114]. Besides, miR-125b-5p and miR-181b-5p may suppress keratinocyte proliferation by targeting fibroblast growth factor receptor 2 (FGFR2) and ubiquitin-specific peptidase 2 (USP2) or TLR4, respectively [115, 116]. Furthermore, miR-20a-3p and miR-330 are two IL-22 responsive microRNAs identified in psoriasis. miR-20-3p promoted keratinocyte cell apoptosis and inhibited cell proliferation by Scm like with four mbt domains 1 (SFMBT1) and subsequent TGF-β1/Survivin pathway [117]. miR-330 can directly target catenin beta 1 (CTNNB1, also known as β-catenin), leading to repression of CyclinD1 and Axin2 and subsequent suppression of keratinocyte proliferation [118]. Importantly, miR-876-5p suppressed PI3K/AKT and ERK signaling way to regulate keratinocyte proliferation by targeting Angiopoietin-1 (Ang-1) [119]. Additionally, as a well-known tumor suppressor, miR-217 targeted Grainyhead-like 2 (GRHL2), a developmental transcriptional factor with ability of influencing epithelial barrier function and keratinocyte differentiation, to inhibit keratinocyte proliferation and promote cell differentiation [120].

**Fig. 3:**
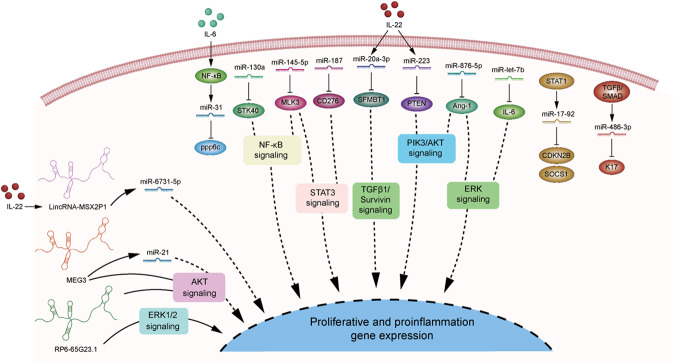
Non-coding RNAs and their involved signaling pathways in psoriasis. Non-coding RNAs play pivotal roles in the pathogenesis of psoriasis. In keratinocytes, they can affect cell proliferation, apoptosis, differentiation, and inflammatory response through targeting multiple signaling pathways, such as NF-κB signaling, STAT3 signaling, ERK signaling, AKT signaling, etc. Also, expression of some of microRNAs are found to be regulated by NF-κB, STAT, or TGFβ signaling. MEG3 maternally expressed gene 3, ppp6c protein phosphatase 6, STK40 serine/threonine kinase 40, MLK3 mixed-lineage kinase 3, SFMBT1 Scm like with four mbt domains 1, PTEN phosphatase and tensin homolog, Ang1 Angiopoietin-1, CDKN2B cyclin-dependent kinase inhibitor 2B, SOCS1 suppressor of cytokine signaling 1, K17 keratin 17.

MicroRNAs play crucial roles in psoriasis, particularly modulating keratinocyte proliferation, differentiation, apoptosis, and inflammation. With all those important signaling pathways involved, microRNAs demonstrate such a comprehensive and potent role in the pathogenesis of psoriasis (Fig. 3).

#### Role of lncRNAs in psoriatic keratinocytes

Unlike microRNAs, lncRNAs are a group of non-protein coding transcripts >200 nucleotides and regulate gene expression at both transcriptional and post-transcriptional levels [121]. Increasing evidence has shown that lncRNAs are involved in the pathogenesis of psoriasis, affecting the function of keratinocytes, T cells and dendritic cells. In this review, we are specially focusing on the role of lncRNAs in keratinocytes.

Psoriasis-associated non-protein coding RNA induced by stress (PRINS), the first identified psoriasis susceptibility-associated lncRNA, was highly expressed in the epidermis of non-lesional and lesional skin of psoriatic patients. PRINS was induced in response to the stress, and silencing of it decreased keratinocyte viability under stress condition [122]. Further investigation identified G1P3 as a downstream target of PRINS, which was also upregulated in psoriatic epidermis and exerted anti-apoptotic effects in keratinocytes [123]. Besides, lncRNA-MSX2P1 was elevated in the lesional skin of psoriatic patients and IL-22-stimulated keratinocytes. It accelerated IL-22-induced keratinocyte proliferation and inhibited apoptosis by inhibition of miR-6731-5p and activation of S100A7 [124]. Similarly, upregulation of lncRNA RP6-65G23.1 has been found in psoriatic epidermis [125]. Overexpression of RP6-65G23.1 promoted keratinocyte proliferation and decreased cell apoptosis, whereas silencing of it showed opposite effects. Of note, RP6-65G23.1 regulated keratinocyte proliferation via AKT and ERK1/2 pathways, and affected keratinocyte apoptosis by Bcl2 and Bcl-xl [121]. In contrast, lncRNA maternally expressed gene 3 (MEG3) was markedly downregulated in psoriatic skin. MEG3 suppressed keratinocyte proliferation and accelerated cell apoptosis by targeting miR-21 and increasing the expression of Caspase 8 [126]. Furthermore, MEG3 inhibited keratinocyte inflammatory response and enhanced autophagy via PI3K/AKT/mTOR signaling pathway in vitro or in vivo [127].

Therefore, lncRNAs influence the proliferation, apoptosis and inflammatory responses of keratinocytes in psoriasis. Identifying dysregulated lncRNAs and the related networks involved in psoriasis will be an important area of research and would provide potential new clues for future diagnosis and treatment of this disease.

### Antimicrobial peptides

Antimicrobial peptides, including LL37, β-defensins and S100 proteins are small proteins that activate innate immune response and are associated with psoriasis pathogenesis [128]. Studied found antimicrobial peptides could also regulate the function of keratinocytes. S100A7 is an antimicrobial peptide that is stored in differentiated keratinocytes and S100A7 was significantly elevated in psoriasis patients. Studies suggest that S100A7 expression can be induced by differentiation of keratinocytes dependent on protein kinase C pathway or downregulation of Caspase 8. However, overexpression of S100A7 led to aberrant keratinocyte differentiation in psoriasis [129–131], suggesting a negative feedback during psoriasis development. Furthermore, C10orf99 is another antimicrobial peptide identified recently that was highly expressed in psoriatic epidermis of patients or IMQ-induced mouse model. Topical application of C10orf99 shRNA effectively attenuated IMQ-induced psoriasis-like dermatitis. Notably, C10orf99 promoted keratinocyte proliferation by enhancing the G1/S transition and activating the ERK1/2 and NF-κB pathways, thus contributing to psoriasis pathogenesis [132]. Therefore, antimicrobial peptides exert important impacts on psoriatic keratinocytes.

### Proteins with other functions

#### Proteins upregulated in psoriasis

Emerging evidence has shown that the ubiquitin-proteasome system plays a crucial role in the pathogenesis of psoriasis. Zieba and colleagues showed that the proteasome assembly chaperone POMP (proteasome maturation protein) was upregulated in psoriatic skin, resulting in an increase of proteasome levels and activities. Silencing POMP inhibited cell proliferation and differentiation, and promoted cell apoptosis via inhibition of the proteasome assembly [133]. E3 Ligase tripartite motif-containing 21 (Trim21) belongs to the Trim protein family with E3 ligase activity. In psoriatic epidermis, Trim21 was found overexpressed and induced activation of STAT3 through ubiquitylating and stabilizing KRT17 in keratinocytes, thus promoting the development of psoriasis [134]. Neural precursor cell expressed developmentally downregulated 4-like (NEDD4L) is another E3 ligase that was downregulated in psoriatic epidermis. Suppression of NEDD4L promoted keratinocyte hyperplasia by mediating GP130 degradation and activation of STAT3 [135]. TRAF6 is a signaling adaptor and E3 ubiquitin ligase. In psoriasis, IL-17 signals through TRAF6, then activates NF-κB and MAPK pathways. Keratinocyte-specific deletion of TRAF6 diminished psoriasiform hyperplasia and IL-17-mediated inflammation [136]. Prokineticin 2 (PK2), a neuroendocrine peptide, is a psoriasis-specific factor highly expressed psoriatic skins. PK2 enhanced the production of IL-1 in keratinocytes and macrophages, thus inducing keratinocyte hyperproliferation and inflammatory cascades in psoriasis. Overexpression of PK2 exacerbated psoriasis-like dermatitis in mice, whereas knockdown of PK2 ameliorated psoriasis-like dermatitis [137]. Therefore, PK2 could be a novel psoriasis-specific target in the treatment of psoriasis. Ras-related C3 botulinum toxin substrate 1 (RAC1) belongs to the small GTPases of the Rho family. RAC1 was hyperactivated in psoriatic epidermis and overexpression of RAC1 in keratinocytes caused psoriasis-like skin lesions in mice. Epidermal activation of RAC1 stimulated a variety of key signaling pathways, such as STAT3, ZNF750, and NF-κB, leading to keratinocyte hyperproliferation and cytokines production, as well as cell differentiation inhibition [138]. Plexin-B2, an axon-guidance molecule, was found greatly increased in keratinocytes of psoriatic patients and IMQ-induced psoriatic mouse model. Silencing Plexin-B2 decreased IMQ-induced psoriatic dermatitis. Mechanically, binding by its ligand CD100, Plexin-B2 promoted the production of inflammatory chemokines/cytokines and the formation of the NLRP inflammasome in keratinocytes through activating NF-κB pathway, subsequently strengthening inflammatory responses of keratinocytes in psoriasis [139]. CCN1, also known as cysteine-rich protein 61 (Cyr61), is an extracellular protein elevated in psoriatic lesions. It stimulated keratinocyte proliferation and a variety of immune-related molecule expression by keratinocytes, such as IL-8, IL1-β, CCL20, HLA-ABC, HLA-DR, and ICAM. Silencing or blocking CCN1 alleviated IL-23- or IMQ-induced psoriasis-like dermatitis [140–143]. High-mobility group protein B1 (HMGB1) is a nuclear protein that can be released to act as a cytokine when cells undergo stress. In psoriasis, HMGB1 can be released from keratinocytes, which potentiates the production and secretion of IL-18 by keratinocytes through an autocrine mechanism. Importantly, blocking HMGB1 or IL-8 by neutralizing antibodies not only attenuated but also accelerated the recovery from psoriasis-like dermatitis induced by IMQ [144]. Recently, a member of the epidermal differentiation complex, Cornulin (CRNN), was shown highly expressed in psoriatic epidermis from patients or IMQ-induced mouse model. Induction of CRNN in keratinocytes activated PI3K/AKT pathway, contributing to keratinocyte hyperproliferation [145].

#### Proteins downregulated in psoriasis

Galectin-3, which belongs to the galectin family of β-galactoside-binding lectins, is a psoriasis-specific protein downregulated in psoriatic epidermis. Galectin-3 deficient in epidermal keratinocytes resulted in a spontaneous development of psoriasis-like phenotype. Administration of recombinant Galectin-3 ameliorated IMQ-induced psoriasis-like dermatitis. Notably, downregulation of Galectin-3 altered keratinocyte differentiation and apoptosis and induced the expression of antimicrobial peptides (S100A7, S100A8, and S100A9) and chemokines (CXCL1, CXCL8, and CCL20) by activation of JNK signaling, leading to neutrophil accumulation [146]. Cholecystokinin octapeptide (CCK8), a stimulatory hormone released from enteroendocrine I-cells of the intestine, was constitutively expressed in the epidermis of normal skin, but decreased in psoriatic patients. Administration of sulfated CCK8 ameliorated IMQ-induced psoriasiform hyperplasia and inflammation through an autocrine or paracrine manner with decreased expression of IL-17, IL-22, and IL-6 but not IL-23. In vitro studies found that IL-17 stimulation reduced CCK8 expression, which may lead to the induction of IL-6 [147]. Connexin 43 (Cx43) is a member of gap junction protein, which is abundantly expressed in epidermis. Cx43 was markedly downregulated in psoriatic epidermis and IL-22-stimulated keratinocyte. Downregulation of Cx43 significantly promoted cell proliferation and decreased gap junction intercellular communication in keratinocytes, resulting from IL-22-induced JNK pathway activation [148].

## Concluding remarks

In this review, we have highlighted the critical roles of keratinocytes in psoriasis. Keratinocytes participate in both the initiation and maintenance phases of psoriasis. There are various factors that can regulate keratinocytes, including genetic regulation, cytokines and receptors, metabolism, cell signaling, transcription factors, non-coding RNAs, antimicrobial peptides, and proteins with other different functions. These modulating factors are not independent, but work together to alter the biological behavior of keratinocytes via multiple mechanisms, linking keratinocytes with psoriasis.

Although our understanding of the role of keratinocytes in psoriatic pathogenesis has advanced considerably, our knowledge about how various factors regulate the detailed functions of keratinocytes are still limited. Right now, many anti-psoriatic drugs, especially the biologics have demonstrated good efficacy, however, long-term efficacy and safety are still the problems for psoriasis treatment. Therefore, it is of great need to discover better treatment targeting keratinocytes more selectively and efficiently. Investigation into the mechanisms of keratinocyte-immune cell interaction network may shed light on restoring keratinocyte homeostasis and benefiting the alleviation of psoriasis.

## Supplementary information

Author Contribution Statement

## Data Availability

All data generated during and/or analyzed during the current study are available.

## References

[1] Rendon A, Schäkel K (2019). Psoriasis pathogenesis and treatment. Int J Mol Sci.

[2] Boehncke WH, Schön MP (2015). Psoriasis. Lancet.

[3] Ni X, Lai Y (2020). Keratinocyte: a trigger or an executor of psoriasis?. J Leukoc Biol.

[4] Dopytalska K, Ciechanowicz P, Wiszniewski K, Szymańska E, Walecka I (2021). The role of epigenetic factors in psoriasis. Int J Mol Sci.

[5] Perera GK, Di Meglio P, Nestle FO (2012). Psoriasis. Annu Rev Pathol.

[6] Greb JE, Goldminz AM, Elder JT, Lebwohl MG, Gladman DD, Wu JJ (2016). Psoriasis. Nat Rev Dis Prim.

[7] Lowes MA, Russell CB, Martin DA, Towne JE, Krueger JG (2013). The IL-23/T17 pathogenic axis in psoriasis is amplified by keratinocyte responses. Trends Immunol.

[8] Nestle FO, Kaplan DH, Barker J (2009). Psoriasis. New Engl J Med.

[9] Hawkes JE, Yan BY, Chan TC, Krueger JG (2018). Discovery of the IL-23/IL-17 signaling pathway and the treatment of psoriasis. J Immunol.

[10] Griffiths CEM, Armstrong AW, Gudjonsson JE, Barker J (2021). Psoriasis. Lancet.

[11] Hawkes JE, Chan TC, Krueger JG (2017). Psoriasis pathogenesis and the development of novel targeted immune therapies. J Allergy Clin Immunol.

[12] Wang Z, Zhou H, Zheng H, Zhou X, Shen G, Teng X (2021). Autophagy-based unconventional secretion of HMGB1 by keratinocytes plays a pivotal role in psoriatic skin inflammation. Autophagy.

[13] Mahil SK, Capon F, Barker JN (2015). Genetics of psoriasis. Dermatol Clin.

[14] Tsoi LC, Stuart PE, Tian C, Gudjonsson JE, Das S, Zawistowski M (2017). Large scale meta-analysis characterizes genetic architecture for common psoriasis associated variants. Nat Commun.

[15] Wang M, Zhang S, Zheng G, Huang J, Songyang Z, Zhao X (2018). Gain-of-function mutation of card14 leads to spontaneous psoriasis-like skin inflammation through enhanced keratinocyte response to IL-17A. Immunity.

[16] Mellett M, Meier B, Mohanan D, Schairer R, Cheng P, Satoh TK (2018). CARD14 gain-of-function mutation alone is sufficient to drive IL-23/IL-17-mediated psoriasiform skin inflammation in vivo. J Invest Dermatol.

[17] Tanaka M, Kobiyama K, Honda T, Uchio-Yamada K, Natsume-Kitatani Y, Mizuguchi K (2018). Essential role of CARD14 in murine experimental psoriasis. J Immunol.

[18] Afonina IS, Van Nuffel E, Baudelet G, Driege Y, Kreike M, Staal J (2016). The paracaspase MALT1 mediates CARD14-induced signaling in keratinocytes. EMBO Rep.

[19] Devos M, Mogilenko DA, Fleury S, Gilbert B, Becquart C, Quemener S (2019). Keratinocyte expression of A20/TNFAIP3 controls skin inflammation associated with atopic dermatitis and psoriasis. J Invest Dermatol.

[20] Ippagunta SK, Gangwar R, Finkelstein D, Vogel P, Pelletier S, Gingras S (2016). Keratinocytes contribute intrinsically to psoriasis upon loss of Tnip1 function. Proc Natl Acad Sci USA.

[21] Harirchian P, Lee J, Hilz S, Sedgewick AJ, Perez White BE, Kesling MJ (2019). A20 and ABIN1 suppression of a keratinocyte inflammatory program with a shared single-cell expression signature in diverse human rashes. J Invest Dermatol.

[22] Wang Z, Liang W, Zhang B, Lv M, Wang J, Zhang L (2008). Single nucleotide polymorphisms of VEGF gene and Psoriasis risk. J Dermatol Sci.

[23] Xia YP, Li B, Hylton D, Detmar M, Yancopoulos GD, Rudge JS (2003). Transgenic delivery of VEGF to mouse skin leads to an inflammatory condition resembling human psoriasis. Blood.

[24] Kunstfeld R, Hirakawa S, Hong YK, Schacht V, Lange-Asschenfeldt B, Velasco P (2004). Induction of cutaneous delayed-type hypersensitivity reactions in VEGF-A transgenic mice results in chronic skin inflammation associated with persistent lymphatic hyperplasia. Blood.

[25] Halin C, Fahrngruber H, Meingassner JG, Bold G, Littlewood-Evans A, Stuetz A (2008). Inhibition of chronic and acute skin inflammation by treatment with a vascular endothelial growth factor receptor tyrosine kinase inhibitor. Am J Pathol.

[26] Jung K, Lee D, Lim HS, Lee SI, Kim YJ, Lee GM (2011). Double anti-angiogenic and anti-inflammatory protein Valpha targeting VEGF-A and TNF-alpha in retinopathy and psoriasis. J Biol Chem.

[27] Benhadou F, Glitzner E, Brisebarre A, Swedlund B, Song Y, Dubois C (2020). Epidermal autonomous VEGFA/Flt1/Nrp1 functions mediate psoriasis-like disease. Sci Adv.

[28] Lambert S, Swindell WR, Tsoi LC, Stoll SW, Elder JT (2017). Dual role of Act1 in keratinocyte differentiation and host defense: TRAF3IP2 silencing alters keratinocyte differentiation and inhibits IL-17 responses. J Invest Dermatol.

[29] Lipovsky A, Slivka PF, Su Z, Wang Y, Paulsboe S, Wetter J (2021). ACT1 is required for murine IL-23-induced psoriasiform inflammation potentially independent of E3 ligase activity. J Invest Dermatol.

[30] Veal CD, Clough RL, Barber RC, Mason S, Tillman D, Ferry B (2001). Identification of a novel psoriasis susceptibility locus at 1p and evidence of epistasis between PSORS1 and candidate loci. J Med Genet.

[31] Hsieh WL, Huang YH, Wang TM, Ming YC, Tsai CN, Pang JH (2015). IFI27, a novel epidermal growth factor-stabilized protein, is functionally involved in proliferation and cell cycling of human epidermal keratinocytes. Cell Prolif.

[32] Madonna S, Girolomoni G, Dinarello CA, Albanesi C (2019). The significance of IL-36 hyperactivation and IL-36R targeting in psoriasis. Int J Mol Sci.

[33] Tortola L, Rosenwald E, Abel B, Blumberg H, Schafer M, Coyle AJ (2012). Psoriasiform dermatitis is driven by IL-36-mediated DC-keratinocyte crosstalk. J Clin Invest.

[34] Mossner R, Wilsmann-Theis D, Oji V, Gkogkolou P, Lohr S, Schulz P (2018). The genetic basis for most patients with pustular skin disease remains elusive. Br J Dermatol.

[35] Mahil SK, Twelves S, Farkas K, Setta-Kaffetzi N, Burden AD, Gach JE (2016). AP1S3 Mutations Cause Skin Autoinflammation by Disrupting Keratinocyte Autophagy and Up-Regulating IL-36 Production. J Invest Dermatol.

[36] Li H, Yao Q, Mariscal AG, Wu X, Hulse J, Pedersen E (2018). Epigenetic control of IL-23 expression in keratinocytes is important for chronic skin inflammation. Nat Commun.

[37] Furue M, Furue K, Tsuji G, Nakahara T (2020). Interleukin-17A and keratinocytes in psoriasis. Int J Mol Sci.

[38] Xu M, Lu H, Lee YH, Wu Y, Liu K, Shi Y (2018). An interleukin-25-mediated autoregulatory circuit in keratinocytes plays a pivotal role in psoriatic skin inflammation. Immunity.

[39] Lauffer F, Jargosch M, Baghin V, Krause L, Kempf W, Absmaier-Kijak M (2020). IL-17C amplifies epithelial inflammation in human psoriasis and atopic eczema. J Eur Acad Dermatol Venereol.

[40] Johnston A, Fritz Y, Dawes SM, Diaconu D, Al-Attar PM, Guzman AM (2013). Keratinocyte overexpression of IL-17C promotes psoriasiform skin inflammation. J Immunol.

[41] Vandeghinste N, Klattig J, Jagerschmidt C, Lavazais S, Marsais F, Haas JD (2018). Neutralization of IL-17C reduces skin inflammation in mouse models of psoriasis and atopic dermatitis. J Invest Dermatol.

[42] Moos S, Mohebiany AN, Waisman A, Kurschus FC (2019). Imiquimod-induced psoriasis in mice depends on the IL-17 signaling of keratinocytes. J Invest Dermatol.

[43] Milora KA, Fu H, Dubaz O, Jensen LE (2015). Unprocessed Interleukin-36alpha regulates psoriasis-like skin inflammation in cooperation with interleukin-1. J Invest Dermatol.

[44] Hernández-Santana YE, Leon G, St Leger D, Fallon PG, Walsh PT (2020). Keratinocyte interleukin-36 receptor expression orchestrates psoriasiform inflammation in mice. Life Sci Alliance.

[45] Muller A, Hennig A, Lorscheid S, Grondona P, Schulze-Osthoff K, Hailfinger S (2018). IkappaBzeta is a key transcriptional regulator of IL-36-driven psoriasis-related gene expression in keratinocytes. Proc Natl Acad Sci USA.

[46] Iznardo H, Puig L (2021). Exploring the role of IL-36 cytokines as a new target in psoriatic disease. Int J Mol Sci.

[47] Guo J, Tu J, Hu Y, Song G, Yin Z (2019). Cathepsin G cleaves and activates IL-36gamma and promotes the inflammation of psoriasis. Drug Des Dev Ther.

[48] Lorscheid S, Muller A, Loffler J, Resch C, Bucher P, Kurschus FC (2019). Keratinocyte-derived IkappaBzeta drives psoriasis and associated systemic inflammation. JCI Insight.

[49] Hashiguchi Y, Yabe R, Chung SH, Murayama MA, Yoshida K, Matsuo K (2018). IL-36alpha from skin-resident cells plays an important role in the pathogenesis of imiquimod-induced psoriasiform dermatitis by forming a local autoamplification loop. J Immunol.

[50] Wang WM, Wu C, Yu XL, Jin HZ (2019). IL-36beta promotes inflammatory activity and inhibits differentiation of keratinocytes in vitro. Chin Med Sci J.

[51] Mahil SK, Catapano M, Di Meglio P, Dand N, Ahlfors H, Carr IM (2017). An analysis of IL-36 signature genes and individuals with IL1RL2 knockout mutations validates IL-36 as a psoriasis therapeutic target. Sci Transl Med.

[52] Nikoopour E, Bellemore SM, Singh B (2015). IL-22, cell regeneration and autoimmunity. Cytokine.

[53] Hao JQ (2014). Targeting interleukin-22 in psoriasis. Inflammation.

[54] Martin JC, Wolk K, Bériou G, Abidi A, Witte-Händel E, Louvet C (2017). Limited presence of IL-22 binding protein, a natural IL-22 inhibitor, strengthens psoriatic skin inflammation. J Immunol.

[55] Sidler D, Wu P, Herro R, Claus M, Wolf D, Kawakami Y (2017). TWEAK mediates inflammation in experimental atopic dermatitis and psoriasis. Nat Commun.

[56] Peng L, Li Q, Wang H, Wu J, Li C, Liu Y (2018). Fn14 deficiency ameliorates psoriasis-like skin disease in a murine model. Cell Death Dis.

[57] Gupta RK, Gracias DT, Figueroa DS, Miki H, Miller J, Fung K (2021). TWEAK functions with TNF and IL-17 on keratinocytes and is a potential target for psoriasis therapy. Sci Immunol.

[58] Huang X, Chen J, Zeng W, Wu X, Chen M, Chen X (2019). Membrane-enriched solute carrier family 2 member 1 (SLC2A1/GLUT1) in psoriatic keratinocytes confers sensitivity to 2-deoxy-D-glucose (2-DG) treatment. Exp Dermatol.

[59] Zhang Z, Zi Z, Lee EE, Zhao J, Contreras DC, South AP (2018). Differential glucose requirement in skin homeostasis and injury identifies a therapeutic target for psoriasis. Nat Med.

[60] Choi SY, Heo MJ, Lee C, Choi YM, An IS, Bae S (2020). 2-deoxy-d-glucose ameliorates animal models of dermatitis. Biomedicines.

[61] Liu YZ, Xu MY, Dai XY, Yan L, Li L, Zhu RZ (2021). Pyruvate kinase M2 mediates glycolysis contributes to psoriasis by promoting keratinocyte proliferation. Front Pharmacol.

[62] Kamleh MA, Snowden SG, Grapov D, Blackburn GJ, Watson DG, Xu N (2015). LC-MS metabolomics of psoriasis patients reveals disease severity-dependent increases in circulating amino acids that are ameliorated by anti-TNFalpha treatment. J Proteome Res.

[63] Dutkiewicz EP, Hsieh KT, Wang YS, Chiu HY, Urban PL (2016). Hydrogel micropatch and mass spectrometry-assisted screening for psoriasis-related skin metabolites. Clin Chem.

[64] Armstrong AW, Wu J, Johnson MA, Grapov D, Azizi B, Dhillon J (2014). Metabolomics in psoriatic disease: pilot study reveals metabolite differences in psoriasis and psoriatic arthritis. F1000Res.

[65] Kang H, Li X, Zhou Q, Quan C, Xue F, Zheng J (2017). Exploration of candidate biomarkers for human psoriasis based on gas chromatography-mass spectrometry serum metabolomics. Br J Dermatol.

[66] Xia X, Cao G, Sun G, Zhu L, Tian Y, Song Y (2020). GLS1-mediated glutaminolysis unbridled by MALT1 protease promotes psoriasis pathogenesis. J Clin Invest.

[67] Zeng C, Wen B, Hou G, Lei L, Mei Z, Jia X (2017). Lipidomics profiling reveals the role of glycerophospholipid metabolism in psoriasis. Gigascience.

[68] Pietrzak A, Chabros P, Grywalska E, Kicinski P, Pietrzak-Franciszkiewicz K, Krasowska D (2019). Serum lipid metabolism in psoriasis and psoriatic arthritis - an update. Arch Med Sci.

[69] Pietrzak A, Michalak-Stoma A, Chodorowska G, Szepietowski JC (2010). Lipid disturbances in psoriasis: an update. Mediators Inflamm.

[70] Nowowiejska J, Baran A, Flisiak I (2021). Aberrations in lipid expression and metabolism in psoriasis. Int J Mol Sci.

[71] Yu Z, Gong Y, Cui L, Hu Y, Zhou Q, Chen Z (2020). High-throughput transcriptome and pathogenesis analysis of clinical psoriasis. J Dermatol Sci.

[72] Reines I, Kietzmann M, Mischke R, Tschernig T, Luth A, Kleuser B (2009). Topical application of sphingosine-1-phosphate and FTY720 attenuate allergic contact dermatitis reaction through inhibition of dendritic cell migration. J Invest Dermatol.

[73] Schuppel M, Kurschner U, Kleuser U, Schafer-Korting M, Kleuser B (2008). Sphingosine 1-phosphate restrains insulin-mediated keratinocyte proliferation via inhibition of Akt through the S1P2 receptor subtype. J Invest Dermatol.

[74] Schaper K, Dickhaut J, Japtok L, Kietzmann M, Mischke R, Kleuser B (2013). Sphingosine-1-phosphate exhibits anti-proliferative and anti-inflammatory effects in mouse models of psoriasis. J Dermatol Sci.

[75] Ji M, Xue N, Lai F, Zhang X, Zhang S, Wang Y (2018). Validating a selective S1P1 receptor modulator Syl930 for psoriasis treatment. Biol Pharm Bull.

[76] Jeon S, Song J, Lee D, Kim GT, Park SH, Shin DY (2020). Inhibition of sphingosine 1-phosphate lyase activates human keratinocyte differentiation and attenuates psoriasis in mice. J Lipid Res.

[77] Luan C, Chen X, Zhu Y, Osland JM, Gerber SD, Dodds M (2019). Potentiation of psoriasis-like inflammation by PCSK9. J Invest Dermatol.

[78] Calautti E, Avalle L, Poli V (2018). soriasis: A STAT3-centric view. Int J Mol Sci.

[79] Woo YR, Cho DH, Park HJ (2017). Molecular mechanisms and management of a cutaneous inflammatory disorder: psoriasis. Int J Mol Sci.

[80] Sano S, Chan KS, Carbajal S, Clifford J, Peavey M, Kiguchi K (2005). Stat3 links activated keratinocytes and immunocytes required for development of psoriasis in a novel transgenic mouse model. Nat Med.

[81] Nakajima K, Kanda T, Takaishi M, Shiga T, Miyoshi K, Nakajima H (2011). Distinct roles of IL-23 and IL-17 in the development of psoriasis-like lesions in a mouse model. J Immunol.

[82] Ravipati A, Nolan S, Alphonse M, Dikeman D, Youn C, Wang Y (2021). IL-6R/signal transducer and activator of transcription 3 signaling in keratinocytes rather than in T cells induces psoriasis-like dermatitis in mice. J Invest Dermatol.

[83] Lizzul PF, Aphale A, Malaviya R, Sun Y, Masud S, Dombrovskiy V (2005). Differential expression of phosphorylated NF-kappaB/RelA in normal and psoriatic epidermis and downregulation of NF-kappaB in response to treatment with etanercept. J Invest Dermatol.

[84] Rebholz B, Haase I, Eckelt B, Paxian S, Flaig MJ, Ghoreschi K (2007). Crosstalk between keratinocytes and adaptive immune cells in an IkappaBalpha protein-mediated inflammatory disease of the skin. Immunity.

[85] Suzuki K, Suzuki K, Yabe Y, Iida K, Ishikawa J, Makita S (2021). NF-κB1 contributes to imiquimod-induced psoriasis-like skin inflammation by inducing Vγ4(+)Vδ4(+)γδT17 cells. J Invest Dermatol.

[86] Sakurai K, Dainichi T, Garcet S, Tsuchiya S, Yamamoto Y, Kitoh A (2019). Cutaneous p38 mitogen-activated protein kinase activation triggers psoriatic dermatitis. J Allergy Clin Immunol.

[87] Mose M, Kang Z, Raaby L, Iversen L, Johansen C (2013). TNFalpha- and IL-17A-mediated S100A8 expression is regulated by p38 MAPK. Exp Dermatol.

[88] Johansen C, Kragballe K, Westergaard M, Henningsen J, Kristiansen K, Iversen L (2005). The mitogen-activated protein kinases p38 and ERK1/2 are increased in lesional psoriatic skin. Br J Dermatol.

[89] Huang X, Yu P, Liu M, Deng Y, Dong Y, Liu Q (2019). ERK inhibitor JSI287 alleviates imiquimod-induced mice skin lesions by ERK/IL-17 signaling pathway. Int Immunopharmacol.

[90] Yang J, Sun L, Han J, Zheng W, Peng W (2019). DUSP1/MKP-1 regulates proliferation and apoptosis in keratinocytes through the ERK/Elk-1/Egr-1 signaling pathway. Life Sci.

[91] Bai J, Liu Z, Xu Z, Ke F, Zhang L, Zhu H (2015). Epigenetic downregulation of SFRP4 contributes to epidermal hyperplasia in psoriasis. J Immunol.

[92] Jia J, Li C, Yang J, Wang X, Li R, Luo S (2018). Yes-associated protein promotes the abnormal proliferation of psoriatic keratinocytes via an amphiregulin dependent pathway. Sci Rep.

[93] Yang L, Fan X, Cui T, Dang E, Wang G (2017). Nrf2 Promotes Keratinocyte Proliferation in Psoriasis through Up-Regulation of Keratin 6, Keratin 16, and Keratin 17. J Invest Dermatol.

[94] Zenz R, Eferl R, Kenner L, Florin L, Hummerich L, Mehic D (2005). Psoriasis-like skin disease and arthritis caused by inducible epidermal deletion of Jun proteins. Nature.

[95] Zolotarenko A, Chekalin E, Piruzian E, Bruskin S (2018). FRA1 mediates the activation of keratinocytes: Implications for the development of psoriatic plaques. Biochim Biophys Acta Mol Basis Dis.

[96] Gordon WM, Zeller MD, Klein RH, Swindell WR, Ho H, Espetia F (2014). A GRHL3-regulated repair pathway suppresses immune-mediated epidermal hyperplasia. J Clin Invest.

[97] Goldie SJ, Cottle DL, Tan FH, Roslan S, Srivastava S, Brady R (2018). Loss of GRHL3 leads to TARC/CCL17-mediated keratinocyte proliferation in the epidermis. Cell Death Dis.

[98] Hawkes JE, Nguyen GH, Fujita M, Florell SR, Callis Duffin K, Krueger GG (2016). microRNAs in psoriasis. J Invest Dermatol.

[99] Liu Q, Wu DH, Han L, Deng JW, Zhou L, He R (2017). Roles of microRNAs in psoriasis: Immunological functions and potential biomarkers. Exp Dermatol.

[100] Bartel DP (2009). MicroRNAs: target recognition and regulatory functions. Cell.

[101] Srivastava A, Nikamo P, Lohcharoenkal W, Li D, Meisgen F, Xu Landén N (2017). MicroRNA-146a suppresses IL-17-mediated skin inflammation and is genetically associated with psoriasis. J Allergy Clin Immunol.

[102] Yan S, Xu Z, Lou F, Zhang L, Ke F, Bai J (2015). NF-κB-induced microRNA-31 promotes epidermal hyperplasia by repressing protein phosphatase 6 in psoriasis. Nat Commun.

[103] Zhang W, Yi X, An Y, Guo S, Li S, Song P (2018). MicroRNA-17-92 cluster promotes the proliferation and the chemokine production of keratinocytes: implication for the pathogenesis of psoriasis. Cell Death Dis.

[104] Xiong Y, Chen H, Liu L, Lu L, Wang Z, Tian F (2017). microRNA-130a promotes human keratinocyte viability and migration and inhibits apoptosis through direct regulation of STK40-mediated NF-κB pathway and indirect regulation of SOX9-meditated JNK/MAPK pathway: a potential role in psoriasis. DNA Cell Biol.

[105] Wang R, Wang FF, Cao HW, Yang JY (2019). MiR-223 regulates proliferation and apoptosis of IL-22-stimulated HaCat human keratinocyte cell lines via the PTEN/Akt pathway. Life Sci.

[106] Zhang W, Yi X, Guo S, Shi Q, Wei C, Li X (2014). A single-nucleotide polymorphism of miR-146a and psoriasis: an association and functional study. J Cell Mol Med.

[107] Taganov KD, Boldin MP, Chang KJ, Baltimore D (2006). NF-kappaB-dependent induction of microRNA miR-146, an inhibitor targeted to signaling proteins of innate immune responses. Proc Natl Acad Sci USA.

[108] Hermann H, Runnel T, Aab A, Baurecht H, Rodriguez E, Magilnick N (2017). miR-146b probably assists miRNA-146a in the suppression of keratinocyte proliferation and inflammatory responses in psoriasis. J Invest Dermatol.

[109] Wu Y, Liu L, Bian C, Diao Q, Nisar MF, Jiang X (2018). MicroRNA let-7b inhibits keratinocyte differentiation by targeting IL-6 mediated ERK signaling in psoriasis. Cell Commun Signal.

[110] Yan JJ, Qiao M, Li RH, Zhao XT, Wang XY, Sun Q (2019). Downregulation of miR-145-5p contributes to hyperproliferation of keratinocytes and skin inflammation in psoriasis. Br J Dermatol.

[111] Tang L, He S, Zhu Y, Feng B, Su Z, Liu B (2019). Downregulated miR-187 contributes to the keratinocytes hyperproliferation in psoriasis. J Cell Physiol.

[112] Jiang M, Sun Z, Dang E, Li B, Fang H, Li J (2017). TGFβ/SMAD/microRNA-486-3p signaling axis mediates keratin 17 expression and keratinocyte hyperproliferation in psoriasis. J Invest Dermatol.

[113] Feng SJ, Chu RQ, Ma J, Wang ZX, Zhang GJ, Yang XF (2017). MicroRNA138 regulates keratin 17 protein expression to affect HaCaT cell proliferation and apoptosis by targeting hTERT in psoriasis vulgaris. Biomed Pharmacother.

[114] Zheng Y, Cai B, Li X, Li D, Yin G (2019). MiR-125b-5p and miR-181b-5p inhibit keratinocyte proliferation in skin by targeting Akt3. Eur J Pharmacol.

[115] Suwanwongse K, Shabarek N (2020). miRNA125b downregulation: a review of the novel paradigm of psoriasis epigenetic regulation. Cureus.

[116] Feng C, Bai M, Yu NZ, Wang XJ, Liu Z (2017). MicroRNA-181b negatively regulates the proliferation of human epidermal keratinocytes in psoriasis through targeting TLR4. J Cell Mol Med.

[117] Li R, Qiao M, Zhao X, Yan J, Wang X, Sun Q (2018). MiR-20a-3p regulates TGF-β1/Survivin pathway to affect keratinocytes proliferation and apoptosis by targeting SFMBT1 in vitro. Cell Signal.

[118] Shen H, Zeng B, Wang C, Tang X, Wang H, Liu W (2017). MiR-330 inhibits IL-22-induced keratinocyte proliferation through targeting CTNNB1. Biomed Pharmacother.

[119] A R, Yu P, Hao S, Li Y (2018). MiR-876-5p suppresses cell proliferation by targeting Angiopoietin-1 in the psoriasis. Biomed Pharmacother.

[120] Zhu H, Hou L, Liu J, Li Z (2016). MiR-217 is down-regulated in psoriasis and promotes keratinocyte differentiation via targeting GRHL2. Biochem Biophys Res Commun.

[121] Duan Q, Wang G, Wang M, Chen C, Zhang M, Liu M (2020). LncRNA RP6-65G23.1 accelerates proliferation and inhibits apoptosis via p-ERK1/2/p-AKT signaling pathway on keratinocytes. J Cell Biochem.

[122] Sonkoly E, Bata-Csorgo Z, Pivarcsi A, Polyanka H, Kenderessy-Szabo A, Molnar G (2005). Identification and characterization of a novel, psoriasis susceptibility-related noncoding RNA gene, PRINS. J Biol Chem.

[123] Szegedi K, Sonkoly E, Nagy N, Nemeth IB, Bata-Csorgo Z, Kemeny L (2010). The anti-apoptotic protein G1P3 is overexpressed in psoriasis and regulated by the non-coding RNA, PRINS. Exp Dermatol.

[124] Qiao M, Li R, Zhao X, Yan J, Sun Q (2018). Up-regulated lncRNA-MSX2P1 promotes the growth of IL-22-stimulated keratinocytes by inhibiting miR-6731-5p and activating S100A7. Exp Cell Res.

[125] Tsoi LC, Iyer MK, Stuart PE, Swindell WR, Gudjonsson JE, Tejasvi T (2015). Analysis of long non-coding RNAs highlights tissue-specific expression patterns and epigenetic profiles in normal and psoriatic skin. Genome Biol.

[126] Jia HY, Zhang K, Lu WJ, Xu GW, Zhang JF, Tang ZL (2019). LncRNA MEG3 influences the proliferation and apoptosis of psoriasis epidermal cells by targeting miR-21/caspase-8. BMC Mol Cell Biol.

[127] Tang ZL, Zhang K, Lv SC, Xu GW, Zhang JF, Jia HY (2021). LncRNA MEG3 suppresses PI3K/AKT/mTOR signalling pathway to enhance autophagy and inhibit inflammation in TNF-alpha-treated keratinocytes and psoriatic mice. Cytokine.

[128] Morizane S, Gallo RL (2012). Antimicrobial peptides in the pathogenesis of psoriasis. J Dermatol.

[129] Ekman AK, Vegfors J, Eding CB, Enerbäck C (2017). Overexpression of psoriasin (S100A7) contributes to dysregulated differentiation in psoriasis. Acta Derm Venereol.

[130] Maurelli M, Gisondi P, Danese E, Gelati M, Papagrigoraki A, Del Giglio M (2020). Psoriasin (S100A7) is increased in the serum of patients with moderate-to-severe psoriasis. Br J Dermatol.

[131] Bhatt T, Bhosale A, Bajantri B, Mathapathi MS, Rizvi A, Scita G (2019). Sustained secretion of the antimicrobial peptide S100A7 is dependent on the downregulation of caspase-8. Cell Rep.

[132] Chen C, Wu N, Duan Q, Yang H, Wang X, Yang P (2018). C10orf99 contributes to the development of psoriasis by promoting the proliferation of keratinocytes. Sci Rep.

[133] Zieba BA, Henry L, Lacroix M, Jemaà M, Lavabre-Bertrand T, Meunier L (2017). The proteasome maturation protein POMP increases proteasome assembly and activity in psoriatic lesional skin. J Dermatol Sci.

[134] Yang L, Jin L, Ke Y, Fan X, Zhang T, Zhang C (2018). E3 Ligase Trim21 ubiquitylates and stabilizes keratin 17 to induce STAT3 activation in psoriasis. J Invest Dermatol.

[135] Liu H, Lin W, Liu Z, Song Y, Cheng H, An H (2021). E3 ubiquitin ligase NEDD4L negatively regulates keratinocyte hyperplasia by promoting GP130 degradation. EMBO Rep.

[136] Matsumoto R, Dainichi T, Tsuchiya S, Nomura T, Kitoh A, Hayden MS (2018). Epithelial TRAF6 drives IL-17-mediated psoriatic inflammation. JCI Insight.

[137] He X, Shen C, Lu Q, Li J, Wei Y, He L (2016). Prokineticin 2 plays a pivotal role in psoriasis. EBioMedicine.

[138] Winge MC, Ohyama B, Dey CN, Boxer LM, Li W, Ehsani-Chimeh N (2016). RAC1 activation drives pathologic interactions between the epidermis and immune cells. J Clin Investig.

[139] Zhang C, Xiao C, Dang E, Cao J, Zhu Z, Fu M (2018). CD100-Plexin-B2 promotes the inflammation in psoriasis by activating NF-κB and the inflammasome in keratinocytes. J Invest Dermatol.

[140] Sun Y, Zhang J, Zhou Z, Wu P, Huo R, Wang B (2015). CCN1, a pro-inflammatory factor, aggravates psoriasis skin lesions by promoting keratinocyte activation. J Invest Dermatol.

[141] Li H, Li H, Huo R, Wu P, Shen Z, Xu H (2017). Cyr61/CCN1 induces CCL20 production by keratinocyte via activating p38 and JNK/AP-1 pathway in psoriasis. J Dermatol Sci.

[142] Wu P, Ma G, Zhu X, Gu T, Zhang J, Sun Y (2017). Cyr61/CCN1 is involved in the pathogenesis of psoriasis vulgaris via promoting IL-8 production by keratinocytes in a JNK/NF-κB pathway. Clin Immunol.

[143] Sun Y, Zhang J, Zhai T, Li H, Li H, Huo R (2017). CCN1 promotes IL-1β production in keratinocytes by activating p38 MAPK signaling in psoriasis. Sci Rep.

[144] Zhang W, Guo S, Li B, Liu L, Ge R, Cao T (2017). Proinflammatory effect of high-mobility group protein B1 on keratinocytes: an autocrine mechanism underlying psoriasis development. J Pathol.

[145] Li C, Xiao L, Jia J, Li F, Wang X, Duan Q (2019). Cornulin is induced in psoriasis lesions and promotes keratinocyte proliferation via phosphoinositide 3-kinase/Akt pathways. J Invest Dermatol.

[146] Shi ZR, Tan GZ, Cao CX, Han YF, Meng Z, Man XY (2018). Decrease of galectin-3 in keratinocytes: a potential diagnostic marker and a critical contributor to the pathogenesis of psoriasis. J Autoimmun.

[147] Funakoshi A, Tatsuno K, Shimauchi T, Fujiyama T, Ito T, Tokura Y (2019). Cholecystokinin downregulates psoriatic inflammation by its possible self-regulatory effect on epidermal keratinocytes. J Immunol.

[148] Liang J, Chen P, Li C, Li D, Wang J, Xue R (2019). IL-22 down-regulates Cx43 expression and decreases gap junctional intercellular communication by activating the JNK pathway in psoriasis. J Invest Dermatol.

