# Supplementary material for: Advances in the pathogenesis of psoriasis: from keratinocyte perspective
Source: Cell Death Dis. 2022 Jan 24;13(1):81. doi: 10.1038/s41419-022-04523-3 (PMC8786887; doi:10.1038/s41419-022-04523-3)
Supplement: Supplementary file 1 — Author Contribution Statement [file 41419_2022_4523_MOESM1_ESM.pdf]

DECLARATION OF CONTRIBUTIONS TO ARTICLE

ADMC

Manuscript Number:	Journal Name:	
CDDIS-21-2801RR	*Cell Death & Disease*	(the 'Journal')

Proposed Title of the Contribution:

Advances in the pathogenesis of psoriasis: from keratinocyte perspective (the 'Contribution)

Author(s):

Xue Zhou, Youdong Chen, Lian Cui, Yuling Shi, Chunyuan Guo (the 'Authors')

For all *CDDis* articles, each person named as an author in the published version must be able to show he or she has contributed substantially to the article.

Authorship credit should be based on 1) substantial contributions to conception and design, acquisition of data, or analysis and interpretation of data; 2) drafting the article or revising it critically for important intellectual content; and 3) final approval of the version to be published. Authors should meet conditions 1, 2 and 3.

Any person who cannot be shown to have made a substantial contribution to the article cannot be listed as an author in the final version. The name of any person who is deemed to have made a minor contribution can, however, appear in the Acknowledgments section of the article.

Please complete the table below to indicate the contributions of all named authors to the manuscript.

Author Full Name:	Specification of Contribution to the Manuscript:
Xue Zhou	organized literature and wrote the manuscript
Youdong Chen	organized literature and wrote the manuscript
Lian Cui	revised the manuscript and checked some important information
Yuling Shi	instructed the structure and revision of the manuscript
Chunyuan Guo	provided the idea of this manuscript and revised the manuscript

Please complete the table below to indicate the contributions of all named authors to the figures.

Figure 1:

In Figure 1, Xue Zhou and Youdong Chen designed the layout and finished the drawing of the figure, Lian Cui improved the details of the figure.

Figure 2:

In Figure 2, Xue Zhou and Chunyuan Guo designed the layout and finished the drawing of the figure, Youdong Chen improved the details of the figure.

Figure 3:

In Figure 3, Xue Zhou and Yuling Shi designed the layout and finished the drawing of the figure, Lian Cui improved the details of the figure.

Figure 4:

Figure 5:

Figure 6:

Signed for and on behalf of the Author(s):	Print Name:	Date:
Chunyuan Guo	Chunyuan Guo	12/12/2021
